# Understanding the Origins and Factors of Burnout in Physical Medicine and Rehabilitation: Grounded Theory Analysis

**DOI:** 10.2196/80499

**Published:** 2026-02-26

**Authors:** Robert Simpson, Eva Cohen, Stephanie Posa, Marina Wasilewski, Anthony Feinstein, Mark Bayley, Larry Robinson, Sarah Munce, Carolyn Steele Gray, Kristina Kokorelias

**Affiliations:** 1 University of Toronto Toronto, ON Canada; 2 Toronto Rehabilitation Institute Toronto, ON Canada; 3 University of Glasgow Glasgow, Glasgow City United Kingdom; 4 Queen's University Kingston, ON Canada; 5 Sunnybrook Research Institute Toronto, ON Canada; 6 Holland Bloorview Kids Rehabilitation Hospital Toronto, ON Canada; 7 Lunenfeld-Tanenbaum Research Institute Toronto, ON Canada

**Keywords:** burnout, physical medicine and rehabilitation, physiatrists, grounded theory, medical culture, residency training, moral distress, health care bureaucracy, emotional labor, stigma

## Abstract

**Background:**

Physician burnout is highly prevalent in Physical Medicine and Rehabilitation (PM&R), but its origins and drivers remain poorly understood.

**Objective:**

This study aims to explore the factors contributing to burnout among Canadian physiatrists.

**Methods:**

Using Charmaz’s Constructed Grounded Theory within a qualitative interpretivist paradigm, we interviewed 30 Canadian physiatrists about their experiences with burnout. Analysis was informed by Cooley’s looking-glass self theory.

**Results:**

Burnout in PM&R in Canada stems from a medical culture prioritizing academic excellence over compassionate care. Canadian physiatrists report shame and self-criticism when unable to meet these high standards. Retrospective accounts from Canadian physiatrists suggest that burnout peaks during residency, where autonomy is low and demands are high. Participants also described feeling unprepared to handle patients’ emotional needs and experiencing moral distress when necessary care cannot be delivered due to systemic barriers. Health care bureaucracy further compounds burnout.

**Conclusions:**

Addressing burnout in PM&R in Canada requires upstream systemic and contemporary cultural change.

## Introduction

### Background

Burnout was originally described as an occupational stress syndrome associated with chronically stressful and emotionally intense work conditions [1]. In health care, burnout has been further classified as having 3 overlapping domains, including emotional exhaustion, diminished sense of achievement, and depersonalization, the latter of which refers to objectification of patients, or loss of compassion [2]. Of concern, burnout in physicians around the world is widespread [3], representing a crisis in health care globally. Physician burnout has significant individual, organizational, and societal costs through worse job performance, workforce attrition, and lower patient satisfaction [4,5].

Among physician specialties in the United States, Physical Medicine and Rehabilitation (PM&R) consistently ranks among the most burned out [6] (odds ratio [OR] 1.30), with only Emergency Medicine (OR 2.53, 95% CI 2.18-2.93), Family Medicine (OR 1.46, 95% CI 1.29-1.65), and Radiology (OR 1.31, 95% CI 1.12-1.54) reporting higher odds of burnout. Burnout affects a significant proportion of PM&R specialists and trainees—ranging from 48% to 62% for specialists and up to 83.3% for trainees [7]. Putative factors contributing to burnout in PM&R physicians come mostly from US survey data, mirror those seen in physicians overall, and do not clarify the specialty-specific causes that drive or sustain burnout [6]. PM&R uniquely focuses on the longitudinal care of people with disabling conditions, aiming to treat impairments, support independence, enhance social participation, and improve quality of life [8]. These philosophical underpinnings are based on a biopsychosocial model of health [9]. This is the core work of PM&R specialists (“physiatrists”) [10].

Existing literature has highlighted several potential contributors to burnout among physiatrists, including high patient load, administrative burden, limited resources [10,11], and may also include the emotional toll of managing patients with chronic conditions [6,11,12], especially those subject to trauma [13]. However, these studies have largely not explored the personal experiences of physiatrists that lead to burnout in PM&R. To our knowledge, only one qualitative study has examined burnout in PM&R, focusing on personal strategies for occupational well-being during the COVID-19 pandemic; however, it used descriptive methods and did not develop a theory explaining burnout in the specialty.

Physiatrists’ work with people affected by disabling long-term conditions, coordination of multidisciplinary care, and prioritizing of functional recovery entails unique stressors not fully captured by burnout theories from other medical contexts [14,15]. This gap highlights the need for a PM&R-specific theoretical framework to clarify the unique stressors driving burnout and guide targeted interventions aimed at improving physiatrists’ well-being and patient care.

### Aims

By capturing the lived experiences and perspectives of Canadian physiatrists, this study aims to provide a comprehensive understanding of the origins and factors contributing to burnout in PM&R through a grounded theory approach.

## Methods

### Design

A qualitative exploratory approach using grounded theory was chosen for its suitability in exploring underresearched areas and generating deeper insights into attitudes, cultures, and social processes [16], specifically Charmaz’s constructed grounded theory [17-19]. Grounded theory as a qualitative methodology is used when the focus is on the process and the creation of theory or a conceptual framework [17,18,20]. In this research, grounded theory was used to construct a conceptual understanding of how physiatrists comprehend and experience burnout. Charmaz’s [17-19] approach to grounded theory emphasizes the coconstruction of meaning between researchers and participants, aligning well with our theoretical grounding in Cooley’s [21] looking-glass self midrange theory. The looking-glass self theory posits that individuals form their self-concepts based on how they perceive others to view them. This idea was instrumental in shaping our analysis of how physiatrists internalize and respond to burnout—through both personal experiences and external reflections from colleagues, patients, health care institutions, and social systems overall [21]. Using the looking-glass self theory, this study explored how Canadian physiatrists’ burnout perceptions are shaped by interactions with and perceived judgments of others. This perspective enriched our grounded theory approach by highlighting relational dynamics such as how social comparison and external validation influence feelings of adequacy or support. It also guided the creation of visual and narrative materials, which complemented narrative data by capturing the interplay between internal experiences and external perceptions central to both the sensitizing theory and our subsequent analysis [22]. Visual materials were developed to reflect scenarios where physiatrists interacted with their environments, colleagues, and patients, illustrating how external judgments and expectations contribute to the emotional and psychological responses to burnout. In this context, the visuals served as a creative tool to depict how social comparisons, external validation, and perceived judgments play a role in shaping physiatrists’ experiences of burnout.

The study received approval from the Sunnybrook Research Ethics Board, ensuring voluntary participation, confidentiality, and the right to withdraw.

### Data Collection

#### Recruitment and Sample

Between September 2023 and March 2024, 30 physiatrists from across Canada were recruited for the study. There are an estimated 501 physiatrists in Canada [23]. Eligibility required English proficiency, current employment as a physiatrist, and willingness to join an online interview. Recruitment used advertisements distributed by the Canadian Association of Physical Medicine and Rehabilitation, outreach by PM&R Division Directors, and via public licensing registries for remote areas. Potential participants received an initial email plus up to 2 reminders. The team monitored recruitment to ensure diversity in age, gender, race, geography, subspecialty, and experience with burnout, expanding outreach to PM&R leaders when needed. Participants provided consent before interviews and received a CAD $50 (US $36.05) Amazon gift card afterward.

#### Methods of Data Collection

Data collection occurred from September 2023 to March 2024 through in-depth qualitative interviews conducted by RS and a trained research assistant EC. Questions were designed to be open-ended, to explore participants’ experiences with burnout in PM&R, with the interviewer paying particular attention to views and actions, as well as emergent areas of potential theoretical interest. Audio and video recordings were made via Zoom (Zoom Communications). The semistructured interview guide, focused on burnout experiences among Canadian physiatrists, is available in Multimedia Appendix 1.

#### Data Analysis

In this study, data analysis began alongside data collection, allowing for constant comparison between new data and emerging categories. This method ensured that each round of data collection was informed by the preceding analysis, allowing the research team to refine interview questions and explore areas that required deeper understanding. We used an inductive and iterative process to derive final insights [18]. Coding was done in multiple stages—open coding to identify initial concepts, axial coding to link these concepts together, and selective coding to integrate them into a coherent framework. First, two researchers open-coded the data in NVivo (Lumivero) using descriptive codes on participant responses [24]. The reviewers then met with the principal investigator to discuss the preliminary coding and developed an inductive codebook that was applied to the transcripts. The looking-glass self theory informed our coding process by encouraging us to attend to moments in the interviews where participants referenced feedback from their work environment—whether from peers, supervisors, or patients—as central to their feelings of burnout or professional fulfillment. This theoretical approach underscored the importance of external perceptions in shaping burnout, thus enriching our analysis of the interplay between personal identity, professional role expectations, health institutions, and systemic pressures. From this, the same researchers engaged in a constant comparison analysis where meetings were held to discuss the common higher-order themes implied by clusters of open codes, leading to the development of axial codes reflecting participants’ insights on the conceptualization and understanding of burnout and experiences with this phenomenon [25,26]. The looking-glass self theory served as a sensitizing concept, framing how burnout is shaped through social interactions in PM&R and guiding the interpretation of nuanced reflections on acknowledgment by self, professional peers, and institutions. Two researchers applied axial coding to transcripts, discussing the process weekly [26]. Once team discussions suggested a preliminary understanding of a theoretical framework, the researchers engaged in a final reflexive process of selective (focused) coding [27]. Selective coding involved the deliberate examination of specific aspects of the preliminary framework within the transcripts to deepen our understanding of the framework, explore relationships between the data, and extract meaningful insights [19]. Categories were identified from comparative analysis, culminating in a core category that encapsulated the study’s main theme [18]. Through team discussions, the researchers were able to extrapolate prominent themes within the data to inform an overarching conceptual understanding [19]. In between the weekly meetings, the researchers went independently back to the data to further refine their ideas against the emerging conceptual framework.

We systematically searched for disconfirming cases that challenged emerging themes, documenting and analyzing them to refine our findings. Analysis and team discussions continued until a conceptual framework achieving theoretical saturation was developed [28], where themes around how physiatrists understood and experienced burnout were consistently repeating, with no new dimensions or variations emerging, and the conceptual framework appeared sufficiently dense to explain the origins and factors sustaining burnout in PM&R [27].

#### Positionality

Our positionality as a team influenced this process, as we were mindful of how our own experiences and professional backgrounds shaped the way we coded and interpreted the data [29]. The lead author, a middle-aged Scots Canadian physiatrist with expertise in mindfulness and compassion, contributed insider insight on burnout in high-stress health care settings, framing it through the looking-glass self theory and emphasizing emotional and relational aspects. An interdisciplinary team, including a PhD-trained rehabilitation scientist and early-career researchers, incorporated structural and institutional factors and reflected on their roles, producing a nuanced, reflexive framework capturing the complexity of burnout in PM&R.

### Ethical Considerations

This study was conducted in accordance with the World Medical Association Declaration of Helsinki - Ethical Principles for Medical Research Involving Human Participants and prospectively approved by the Sunnybrook Research Ethics Board (project ID: SUN-5835). Informed consent was obtained electronically from all study participants after the nature and possible consequences of the study were explained. Respect for individual autonomy was emphasized, including that even following the granting of informed consent, participants could choose to withdraw from the study for any reason, without needing to give a reason and without adverse consequence. All participants who provided a semistructured interview received a CAD $50 (US $36.05) Amazon gift card in compensation. All reasonable efforts were made throughout to protect participant safety, dignity, privacy, and confidentiality. All personal identifying information was deidentified, and any interview quotes with potentially identifying information were not included in the study findings.

## Results

### Participant Characteristics

Most participants were women (19/30, 63%), with a mean age of 45.5 years. Most participants were recruited from Ontario (18/30, 60%). The most common racial identity was White European (12/30, 40%), followed by White North American (9/30, 30%), East Asian (6/30, 20%), South Asian (2/30, 7%), and Latin European (1/30, 3%). Participants worked in a wide range of PM&R settings, but most worked in an urban setting (28/30, 93%), full time (22/30, 73%), with 19 (63%) participants working 50 or more hours per week, with mixed inpatient and outpatient populations (24/30, 80%), and in most cases in neurological rehabilitation (13/30, 43%). No PM&R residents were interviewed (Table 1).

**Table 1:** Sociodemographic characteristics of participants.

Characteristic			Values (N=30)
**Sex/gender, n (%)**			
	Female/woman	19 (63.3)	
	Male/man	11 (36.7)	
Age (years), mean (SD)			45.5 (10.5)
Number of years practicing, mean (SD)			13.6 (10.7)
**Province of practice, n (%)**			
	Ontario	18 (60)	
	British Columbia	4 (13)	
	Quebec	3 (10)	
	Saskatchewan	2 (6.7)	
	Alberta	1 (3.3)	
	New Brunswick	1 (3.3)	
	Nova Scotia	1 (3.3)	
**Race/ethnicity^a^** **, n (%)**			
	White European	12 (40)	
	White North American	9 (30)	
	East Asian	6 (20)	
	South Asian	2 (6.7)	
	Mixed	2 (6.7)	
	Latin European	1 (3.3)	
**Specialty^a^** **, n (%)**			
	Neurorehabilitation	13 (43.4)	
	Electromyography	9 (30)	
	Other	8 (26.7)	
	Spinal rehabilitation	5 (16.7)	
	Musculoskeletal	4 (13.3)	
	General rehabilitation	3 (10)	
	Pain	3 (10)	
**Primary professional role, n (%)**			
	Clinician, full-time	22 (73.3)	
	Clinician, part-time	8 (11.8)	
**Secondary professional role^a^** **, n (%)**			
	Clinician-teacher	14 (46.7)	
	Clinician-scientist	13 (43.3)	
	Administrative roles	10 (33.3)	
	Clinician-quality improvement	1 (3.3)	
**Hours worked per week, n (%)**			
	50+	19 (63.3)	
	30-40	7 (23.3)	
	40-50	4 (13.3)	
**Time spent providing virtual care, n (%)**			
	0%-25%	27 (90)	
	25%-50%	3 (10)	
Provides out-of-hours^b^, n (%)			18 (60)
**Clinic setting, n (%)**			
	Mixed inpatient and outpatient	24 (80)	
	Outpatient	6 (20)	
**Rurality, n (%)**			
	Urban	28 (93.3)	
	Urban and rural	2 (6.7)	

^a^Participants could choose more than one option. Percentages may not add up to 100.

^b^Reflects the number and percentage of participants answering “yes” to this question.

### Main Findings

**Figure 1:**
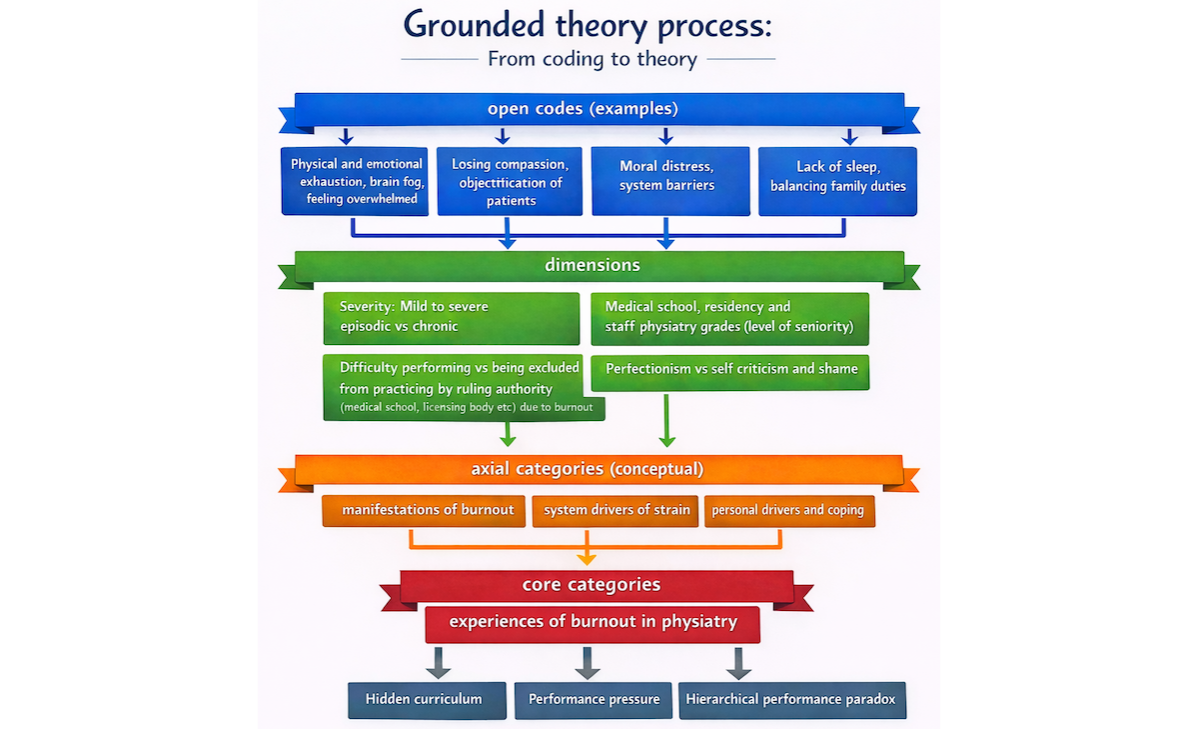
Grounded theory process.

**Figure 2:**
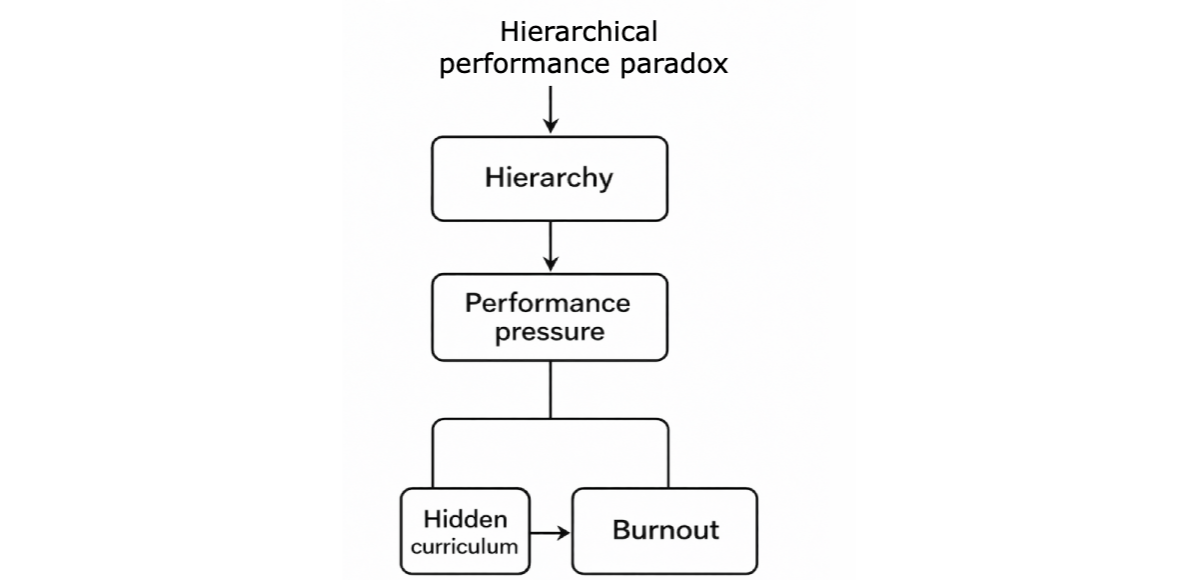
The hierarchical performance paradox and burnout in physiatry.

The study findings present a conceptually grounded theory titled “Burnout in Physical Medicine and Rehabilitation is the Hierarchical Performance Paradox.” The theory comprises 3 interconnected concepts: hierarchical structure, perception of performance, and their interaction in PM&R. In this theory, which is based on retrospective accounts of the lived experience of practicing physiatrists, the origins of burnout in PM&R are sown early in the training of budding physiatrists. Specifically, the hierarchical structure of medical training and practice imposes escalating demands amidst constrained autonomy as Canadian physiatrists progress through their careers, whilst simultaneously the perceived necessity of perfection in performance—shaped by internalized appraisals by peers, patients, and regulatory authorities—foments feelings of imposter syndrome. This hidden curriculum prioritizes performance proficiency above all else, leading to a mindset of “Doctor first, human second” (Woman, aged 33 years) (the hidden curriculum here refers to the implicit lessons, values, and attitudes conveyed through the behaviors and expectations of mentors, colleagues, and the health care system, which are not formally part of the medical educational syllabus [30]). Burnout risk appears highest when autonomy is low and stress is high, fostering self-criticism and shame. Social comparison and perfectionism are reported by Canadian physiatrists to begin in medical school and intensify during PM&R residency, when autonomy and social support decline, and burnout peaks. Later, increased autonomy does not eliminate risk, as expanded responsibilities, emotional demands, health care system inadequacies, and bureaucratic pressures perpetuate moral distress and burnout throughout a Canadian physiatrist’s career. Illustrative quotes from participants are woven throughout the text. Figure 1 depicts the grounded theory process from coding to theory for burnout in Canadian physiatrists. Figure 2 depicts the hierarchical performance paradox.

### The Role of Hierarchy in Burnout in Physical Medicine and Rehabilitation

Retrospective accounts from practicing Canadian physiatrists indicate that burnout in PM&R originates from a hierarchical and performance-driven medical education system that inadvertently de-emphasizes compassionate care, leading to stress and emotional exhaustion:

Medicine is the stodgiest, most conformist profession that there is, and it has not adapted or evolved to the 20th century, and I think that creates a burnout environment.

Man, aged 41 years

Hierarchy in this context refers to the structured levels of authority and responsibility within medical education and practice, where individuals at lower levels, such as medical students and residents, are subject to the oversight, evaluation, and directives of those higher in the system, such as attending physicians and administrators. This hierarchical framework prioritizes academic performance, efficiency, and adherence to institutional norms over compassionate care, creating an environment where Canadian physiatrists face intense pressure, reduced autonomy, and a heightened risk of burnout. A hidden curriculum in this context perpetuates these values, fostering emotional detachment and prioritizing efficiency and exceptional levels of academic performance. These factors, together with systemic constraints and relentless vertical oversight from within the hierarchy, and horizontal peer appraisal, contribute to burnout outcomes such as emotional exhaustion, depersonalization, and a diminished sense of achievement among many Canadian physiatrists.

Participant accounts indicated that the motivations for becoming a physiatrist are largely altruistic, reflecting core values of improving quality of life for people with disabling long-term conditions:

I try my hardest to be compassionate to patients because that’s why I wanted to get into what I wanted to do in physiatry. I feel that you have a longer relationship with people, so you need more of a compassionate approach versus a one-off here and there if you're doing surgery or something like that.

Woman, aged 35 years

However, participants also stressed that, in spite of altruistic motivations, the reality is that performance stratification determines who is eligible to apply for admission to medical school, emphasizing academic performance over other qualities:

I think being in medicine and needing the grades you need to get into medical school, things like that require a certain degree of perfectionism, so it’s something that’s been valued throughout your life.

Woman, aged 35 years

Participants iterated that once admitted to medical school, proficiency in academic performance is strongly emphasized. Failure to meet the bar is associated with adverse social comparisons, shame, and self-criticism:

We’re moving away from that shame-based teaching environment, but we still have a lot of that within our clinical training. Shame-based learning is by no means gone and a lot of the times it's just under the surface rather than directly somebody yelling at you for not being good enough.

Woman, aged 33 years

Compassion was described as not being a primary or explicit focus in this milieu; rather, the emphasis was on knowledge of disease and learning how the system really works:

Yes, I think part of it that I never learned that [self-compassion], it’s not part of training and medicine training magnifies mistakes. We have whole rounds about how you learn from how people, quote unquote, screwed up. So, I think that that’s part of it, is that the culture of medicine doesn’t necessarily lend itself to that.

Man, aged 39 years

Participants recalled how, upon graduating from medical school, as residents in PM&R, they often faced excessive workloads fulfilling the demands stipulated by their superiors, sacrificing their autonomy and sense of self:

I was a resident, so I had no control over. I was just told how much call I needed to take. It was different back then, there was payroll, which is the governing body to the residents, but I don't think it was as strict as it is now. The residents were fairly powerless

Woman, aged 48 years

Based on participant recollections, autonomy appeared inversely related to hierarchy within the medical education and practice they experienced. In a hierarchical structure such as this, individuals at lower levels, that is, residents, have limited control over their workload, schedules, and decision-making, as these are dictated by superiors. This lack of autonomy was described as resulting in residents prioritizing institutional demands over personal needs or values, leading to a diminished sense of self.

Only upon becoming fully qualified did a sense of autonomy return, at which point physiatrists in Canada often assume a leadership role in the multidisciplinary team:

How I really got better was I stopped being a resident and then I became a staff, and I had more autonomy. I think that is a factor to relieving burnout, being able to control your work schedules. Being able to say yes to some things and no to other things. I think that’s actually how it got helped

Woman, aged 37 years

However, participants recounted that the reality is that the job continues to be dictated by hierarchical health systems, regulatory body oversight, and peer appraisal of performance:

But I’ll say that even at [rehab hospital] there have certainly been instances where I feel like the response from the organisation in response to something that happened with an error that then caused harm was not, I think the kindest and most compassionate response

Woman, aged 37 years

Indeed, as Canadian physiatrists advance in their careers and move up the hierarchy, although in one sense autonomy is greater, on the other hand, their responsibilities and expectations also increase, creating a persistent tension between autonomy and normative performance standards.

Regardless, Canadian physiatrists of all stages struggle to provide the care they believe is necessary for their patients due to health system constraints and fear authoritarian reprisal for failing to meet expected high performance standards:

I know that in Ontario, people are always like, don’t mess with Ontario College of Physicians because it’s a big problem.

Woman, aged 32 years

Where burnout is concerned, regulatory bodies were seen as threatening due to their ability to “take away your license” to practice medicine:

The College of Physicians was not chill about it though. Because that was PGY2, right, and then when I graduated, you had to answer some questions about did you ever get time off because of this?...the College of Quebec they were just like, have you or are you in a situation where this will be a problem? There was no differentiating it and then they were like, we need a letter from your doctor saying that you are up to practise medicine

Woman, aged 32 years

This highly stressful situation may partly explain the difficulties Canadian physiatrists face in providing care in this hierarchical practice climate.

And now they’re looking at even things like being rude in your private life as being a reportable offense. I mean, really? 24/7, we have to be models of perfection, or we could be reported and lose our livelihood, which would impact whether we can pay our bills. It’s from all sides. To the point where I struggle with whether or not to encourage my own children to enter the profession. Yes.

Woman, aged 49 years

But the burden of care, I think more and more what happens earlier on is your lifeline is that licence to practise, and if that gets taken away, which seems always to be this looming threat, that if I get caught doing something that’s not according to the College’s mandate or whatever then basically I can’t earn a living because my licence is off.

Man, aged 60 years

Participants recalled that, as medical students, despite altruistic motivations for entering PM&R, low hierarchical status, limited autonomy, and strict academic expectations fostered vulnerability and feelings of inadequacy, contributing to early burnout experiences:

Again, obviously I'm a woman so I have a bit of a lens there, but imposter syndrome, which I think is more common in women, but I still feel…

Woman, aged 35 years

Participants described that medical schools exert authority over those who were underperforming, dictating eligibility for module completion or exclusion from training. Self-care was not explicitly emphasized and seen as antithetical to the culture of medicine:

And rather than being harsh towards yourself when you make mistakes or whatever, it’s being non-judgmental and kind to yourself pretty much…Well that’s definitely not a part of medicine.

Man, aged 39 years

Well, I'm saying that I don't think that there is a curriculum in medicine that teaches people self-compassion and work-life balance.

Woman, aged 58 years

Participants described how this scenario continued into their PM&R residencies, where, as trainees, they had to meet performance standards based on long working hours and antisocial working patterns. These demands prevented them from spending time with friends and family, exercising, and attending to diet and sleep. Autonomy became restricted due to strict shift patterns, and as residents, they remained dependent on guidance from their superiors. During this stage, participants described how burnout loomed large as an ever-present threat:

I did burn out in PGY2 and that was the reason I left, is when I had no more compassion for my patients, like I had no empathy for them. I just didn’t care. I think it was more me being exhausted and other things and then when my patients started pissing me off to be honest. It was just whenever they would talk to me about things, I was like, why are you complaining about this.

Woman, aged 32 years

Participants described that as they transitioned from being a trainee to becoming a fully qualified physiatrist, they took on a greater responsibility and became exposed to greater distress in their patients, “Being a physician, especially for a long time, especially dealing with nasty stuff, I think, if you do let that stuff get to you, it’s going to be really hard to succeed in the long run” (Man, aged 41 years), and increasingly aware of health care system limitations, which was a source of moral distress:

I want to be compassionate for the people in front of me, whatever role I’m having, whether it’s for students and residents or whether it’s for the patients and families. But I’m coming up against either structural or system or contextual issues that don’t let me do the thing that I think is the right thing to do, and that causes distress

Woman, aged 43 years

Risk of burnout was described by participants as ever-present as they struggle to balance growing personal and professional responsibilities. Constant oversight and stringent reporting requirements were seen to create a culture prioritizing protocols and metrics over patient-centered care, participants perceiving institutions to prioritize efficiency over quality in interpersonal interactions:

And when you’re dealing with human beings, that’s extremely difficult to individualize a program for somebody in a system where you’re actually a datapoint.. success is measured by the number of patients sometimes just seen, not even healed, not even heard, not even spoken to.

Woman, aged 49 years

The chronic lack of resources, including inadequate staffing and time constraints, was described by participants as leaving them feeling pressured to prioritize logistical demands over holistic patient needs. Heavy administrative duties and systemic constraints were seen to undermine emotional openness and sensitivity to patient distress. In this context, participants described how they may come to view compassionate care as ancillary rather than a core element:

I think it’s like going over and beyond for patients. And also, being there for them emotionally, even though that’s not really part of your job description, but sometimes they just need a Kleenex and need somebody to give them a hug.

Woman, aged 37 years

Consequently, as Canadian physiatrists’ advance in their careers within such a framework, there may be a gradual erosion of emphasis on compassionate care in favor of meeting normative peer appraisal, health care institution, and regulatory demands. Institutional wellness initiatives were perceived as misdirected, emphasizing personal responsibility for burnout and failing to address core cultural and systemic issues:

We can exercise, we can mediate. We know those things. Those are not the things that we need changes on. We’ve all made those changes already. We need the help in the change in the system. They’re the system factors that get us down

Woman, aged 49 years

### The Hidden Curriculum and Its Damaging Effects in Physical Medicine and Rehabilitation

A hidden curriculum, rooted in medical school and reinforced through professional role modeling, was described by participants as shaping behaviors and attitudes that can drive burnout and erode well-being across a physiatrist’s career. Such a hidden curriculum was seen as subtly prioritizing professional achievement over compassion, system efficiency over patient-centered care, and physical treatments over addressing emotional distress.

I think compassion is really a hidden curriculum kind of thing, and depending on who you’re exposed to, you might or might not get any direct instruction on how it is to be compassionate to people. [Compassion] is not really emphasized a lot in medicine compared to clinical expertise. Our recruitment process and training is directed around shutting down self-compassion.

Woman, aged 33 years

These implicit lessons were described as conflicting with the altruistic motivations that draw many physiatrists into medicine:

Yes, of course. If you're a human being, you're going to be emotionally affected by other people's struggles, your patients' struggles. And that's why you end up in medicine, right, because you really want to help people.

Man, aged 45 years

Participants described that in the hidden curriculum, experiencing burnout is seen as a sign of weakness. To be successful in this arena, participants described how they must meet peer and professional standards at all costs:

And then, some of that worry about what other clinicians are going to think about my decision making and choices, and how that’s going to affect long-term relationships, both with the patient, but also with the other clinicians in the workplace.

Woman, aged 33 years

The stakes were described as high, margins for error small, and risk of shame apparent:

…If we mess up, it’s a big deal…the culture of we expect a lot from our students and learners in, I find it’s sometimes not realistic.

Woman, aged 32 years

Indeed, participants recounted how to meet the demands of their work, they often had to sacrifice parts of themselves:

I definitely have at different times throughout my career. Having relatively recently finished residency, trying to balance work as well as studying for the [regulatory board] exam. Trying to have any semblance of social life and I mean that very loosely, seeing your parents every now and then. It’s not like I see my friends or do anything really for myself all that often.

Woman, aged 35 years

Maintaining this level of professional performance was described as exhausting, physically and emotionally, and to deal with this, physiatrists take steps to minimize the stress they are exposed to in their work.

Participants also described that, to be a successful physiatrist in Canada, this often involved maintaining emotional distance from their patients to avoid burnout and manage the high demands of the profession:

I find those very challenging because I’m just trying to sling through and get through my day treating physical problems. But then when you have these mental problems that make it more challenging to treat the physical problem, I think that takes a lot of time and effort and just makes your job more difficult and it requires more empathy which is draining.

Man, aged 32 years

And, although participants recognized that distress is both physical and emotional, many did not feel they had been well prepared through their medical education to deal with emotional distress prevalent among their patients:

I was downright angry at my medical education early on.. we never even talked about stuff like this, let alone how feelings and emotions get in the way of healthcare.

Man, aged 60 years

I am generally treating physical problems and often times peoples’ psychological distress or mental health problems can worsen their physical symptoms and I just, that’s not a population that I do well with.

Man, aged 32 years

Thus, physiatrists may view emotional distress as not within their purview, leading to compartmentalization of care:

And I think a lot of my colleagues, especially on the outpatient side in Canada, end up seeing a lot of run-of-the mill chronic pain, which ends up being people complaining a lot. I don’t have to see a lot of that and so that helps me, because I actually see real suffering. Not real suffering, psychological suffering is still real, but I’m not trained to deal with the psychological suffering of things like chronic pain.

Man, aged 41 years

As such, Canadian physiatrists may become desensitized to the emotional needs of themselves and their patients, focusing primarily on efficiency and protocolized medicine. However, burnout can still result from the forces of this hidden curriculum, and results can be stark. For some, there is frustration and anger towards the system, as they feel there is no time to be compassionate due to the overwhelming demands of the job,

So, the things that get me burnt out would be like when I have a huge amount of work in a short period of time and I don't know how I'm going to be able to get it done. A lot of work and no time, and uncertainty about how I'm going to be able to get through.

Man, aged 48 years

Others might become disenfranchised completely, adopting the belief that fixing the broken health care system is not their responsibility:

No, I have no interest in doing that. It’s too much of an uphill battle.. I would have to put all of my other interests and hobbies in life aside and have to focus on mobilising myself to participate in the process of improving Canadian healthcare, which I am completely unwilling to do.

Man, aged 41 years

### Physiatrists Lead Multidisciplinary Teams and Focus on Adaptation Rather Than Cure

Participants in this study described the work that they do in PM&R as about leading teams, with a focus on adaptation rather than cure, a source of optimism, enthusiasm, and reward for physiatrists, with many iterating that this was where they derived most joy in their work, and felt most able to help:

I think that’s why rehab is so rewarding. I know that [short-term wins] make a big difference… It’s often those little extra moments that you take.

Woman, aged 34 years

Participants emphasized helping patients adapt to disabling conditions and improve quality of life, rather than “fix” a diagnosis. Participants described that involving patients in their care fosters control and confidence but requires physiatrists to have a deep understanding of individual circumstances, often eliciting profound and painful empathy in the process.

Really severe impairments. Patients that are so injured or have terrible conditions that are very debilitating. It’s very easy to find compassion and empathy for those people. When you also see people who lose a limb, or have a, are paralysed or something. It’s very easy to build your compassion up and feel for that person because it’s terrible. It’s subjectively an awful thing.

Man, aged 32 years

I also try not to absorb the terrible situations all the time and say it's a terrible situation, but I think subconsciously, because I see it all the time and they’re all terrible situations. Usually people that are coming to our services are not coming because of a good situation.

Woman, aged 47 years

However, using the knowledge derived from a deep understanding of patient distress, participants described how, as physiatrists, they sought to create tailored care plans that optimize functional independence in their patients. This holistic approach was seen as fundamental to the conceptualization of PM&R, but also exposes physiatrists to difficult emotions:

I think the patients often express frustration in different ways… It’s not as glamorous or as immediately effective as they had anticipated. And so, that often comes out to us as, ‘why aren’t you fixing this and why is that not working?

Woman, aged 47 years

Participants spoke about the need to boundary themselves in relation to the emotions of others, and to see the limitations of the health care system in which they work with realism and detachment, otherwise they might be quickly overcome by cynicism and burnout. Indeed, participants perceived that forbearance was expected from them in the face of abuse and that, in the predominant culture, their emotional well-being was not a priority:

We take a lot of verbal, emotional, sometimes even physical abuse. The expectation is often to just keep providing care and keep being compassionate. But that’s not so simple in practice. I do find that can be a real challenge.

Woman, aged 34 years

We should be, somebody, at the end of the day has said, you know what, I heard about that patient, it was rough. How are you doing? That must be a really tough thing that you had to deal with, how are you feeling? I think that everybody just presumes that they’re… Everybody else is stressed out, how is that any different than yours.

Woman, aged 47 years

Breaking bad news was another area of PM&R, which participants described as perennially difficult in an emotional sense, especially when patient and family expectations were unrealistic, or were not met, and this experience undermined physiatrists’ sense of proficiency:

I think sometimes [patients] want a magic pill to make all their problems go away without any side effects of any work on their part… That is that part that I find the hardest. When you strive to go above and beyond and give people ten things they can do but they have excuses… they don’t ever get better.

Woman, aged 35 years

As physiatrists coordinate and guide multidisciplinary specialist rehabilitation teams, including deliberating over access to care, participants described how they could often be a focal point for venting from other health care providers:

Eventually my team pushed me to act on it, like could you do something to potentially protect her, but me knowing that there really isn’t an ethical or legal way to go about making this decision on behalf of the patient, I felt very uncompassionate towards my team in that situation.

Man, aged 35 years

Participants also spoke about a sense of not being appreciated broadly, by patients, institutions, system leaders, and even peers. They described how feeling unappreciated by others left them feeling isolated, disconnected, and disillusioned, “Nobody, from a physiatry perspective, has said ‘we appreciate what you do.’ For that reason, I’ve tended to work more collaboratively with my colleagues in other specialties rather than my own colleagues here, which is maybe unhealthy” (Woman, aged 47 years), and disillusioned with the health care system at large. When feeling this way, participants were less inclined to listen to the nuances in patient stories and more inclined to objectify patients:

seeing them as their diagnosis. Like, oh that’s the cervical myelopathy, and seeing it as their list, the tick list of things that you've got to do on rounds, to follow up and you’re just doing it to make sure that you've done your job.

Woman, aged 47 years

### Bureaucracy Alongside Hierarchy

Bureaucracy and hierarchy were described by participants as contributing to burnout in PM&R by shifting focus from patient care to rules, paperwork, and complex approval processes, particularly for disability income support. This pervasive administrative burden was felt to foster powerlessness, frustration, and demoralization, subtly eroding autonomy and engagement more insidiously than direct authoritarian control.

Stupid rules that make no sense and that take a lot of time and effort to work around. So, dealing with the bureaucracy of stupid rules... And that causes a lot of frustration and wasted energy that could be used on other things. Stupid rules... we need to have some ways to be able to say, some direct lines to be able to call somebody and say, this is why this one shouldn’t follow the rules and have someone at the other end being able to say okay. I get it. Yes.

Woman, aged 55 years

Participants perceived rigid rule enforcement as nonsensical, adding unnecessary distress to patients and physiatrists alike. In this context, participants described feeling disconnected from their initial sense of purpose, their professional identity overshadowed by the sheer volume of administrative work, which they felt obliged to do in their derived social and professional roles:

Oh, I think the paperwork. So, usually, rehab forms or disability forms are at least six to ten, maybe 20 pages long.

Woman, aged 49 years

This chronic exposure to perceived impersonal and inflexible bureaucratic hurdles, rather than direct authoritarian mandates, appeared to truly wear down physiatrists, contributing significantly to burnout:

Certainly, the burden of documentation, trying to get down all the documents and organize, sometimes some people, they’ll need forms filled out, and those can be very time-consuming and at times frustrating. Because you feel like you’re writing down things that are already in the record but you have to write them down for the completion of a form for a referral or completion of a form for a disability tax credit, I think documentation definitely.

Man, aged 61 years

## Discussion

### Summary of Main Findings

Using a constructed grounded theory approach, based on retrospective accounts of lived experience among Canadian physiatrists, this study developed a conceptual model of burnout in PM&R, highlighting how medical hierarchy—both explicit and via the hidden curriculum—insidiously shapes professional identity from medical school throughout the practice of PM&R. Unrealistic performance standards, emphasis on academic achievement over compassion, and lack of self-care training erode well-being, foster shame, and limit Canadian physiatrists’ ability to respond empathetically. Coupled to this, excessive paperwork, complex administrative processes, and strict regulatory compliance wear Canadian physiatrists down over time.

While PM&R’s focus on adaptation and holistic care is fulfilling, burnout undermines Canadian physiatrists’ capacity to lead multidisciplinary teams effectively, negatively impacting both colleagues and patients.

### Comparison With Existing Literature

No previous studies have used grounded theory to explore burnout in PM&R. However, findings from previous studies using grounded theory to explore burnout in health care providers share similarities with ours. For example, studies in primary care and stroke medicine also suggested systemic issues such as excessive workloads, shifting job roles, and misalignment between personal and institutional values, lead to burnout [31,32]. Likewise, professional dissonance in these contexts also led to feelings of demoralization and a sense of being undervalued [31]. Among nurses, distressing relational aspects of health care also featured in conceptualizations of burnout, particularly during personal interactions, shaping care provided [33], and social support played a mitigating role, affecting emotional responses and some components of attitudinal reactions [33]. In our study, Canadian physiatrists highlighted how they felt ill-prepared for the emotional demands of the work of PM&R, and professionally isolated amid a culture of expected forbearance in the face of emotional distress, where support for their own distress was limited.

Limited literature addresses the origins and sustaining factors of burnout in PM&R. A recent US qualitative study identified similar themes to ours, including the impact of perfectionism, stigma around disclosing distress, and burdensome administrative tasks. Participants advised younger physiatrists to maintain “agency,” set boundaries, and practice self-compassion to prevent burnout. These comparisons are interesting but derive from the unusual context of the COVID-19 pandemic, and are not in relation to origins or factors sustaining burnout in Canadian physiatrists [34].

With regards to the overarching concept identified in our study, of hierarchy in medicine, this is a well-recognized phenomenon [35], but has not been described previously in relation to burnout in PM&R. Hierarchy in medicine has been described as a means of imbuing power to make and enforce decisions based on knowledge and role that allows for autonomy and self-governance in the profession. Hierarchies in medicine can be conceptualized in both negative, “dysfunctional,” and positive, “functional,” terms. In dysfunctional medical hierarchies, those of lower status in the hierarchy are subject to lower health [36], trainees lack psychological safety and agency, and can be subject to outright abuse. In this way, hierarchies can be harmful, not just to those in low-status positions, that is, medical students, residents, but also to patients, collaboration among multiprofessional teams, which is highly relevant in PM&R.

However, it is important to note that hierarchy may also be beneficial, at the macro level, with enhanced performance and evolvability of modular systems [37], but also for the meso (institution, team), and micro (individual) levels, with role and expectation clarity, enabling a social order that serves a greater purpose, which can support a culture of efficiency, and patient safety where the most responsible clinician is the one making critical decisions regarding patient care, particularly when risk is high, and time is pressured. However, to do this in a way that supports social justice and compassionate care likely requires openness and clarity around normative values and conscious and accountable oversight [38]. If not, hierarchy can also be the basis upon which a hidden curriculum, that is, social norms, can develop, including those around punishment, reward, and progression upwards within the hierarchy. Indeed, medical students appear to actively seek to decipher which behaviors their supervisors value most and to preferentially cultivate these [35]. The implications for the development of compassion, or less desirable behaviors [39], at the individual and organizational levels thus seem clear.

Our theoretical framework, the looking-glass self, provided a helpful lens to explore the origins and factors sustaining burnout in PM&R. Physiatrists in our study relayed how the culture of academic elitism in medicine can lead to adverse social comparisons, imposter syndrome, self-criticism, and shame. In keeping with the looking-glass self precept, Gilbert defines shame as a form of undesired self and a form of social disconnection, but differentiates self-shame from social shame perceived to arise from the judgment (and rejection) of others [40]. Unfortunately, shame is a well-described phenomenon in medical education, which can relate to mistreatment, substandard academic performance, social comparisons, and can be reinforced by hidden curricula. Bynum [41] suggests that shame within medical education can be prevented by explicitly eliminating shame-based teaching strategies, creating a culture of inclusion, and facilitating a mindset of growth in learners. Among internal medicine trainees in the US, exposure to a hidden curriculum (unprofessional conduct, including humiliation by colleagues and patients, disrespectful behavior towards patients) has been found to correlate significantly with all 3 core domains of burnout [42]. Indeed, research into hidden curricula in medical education has previously identified that assessment criteria (which drive what medical students see as being prioritized) do not emphasize compassion as a competency being assessed [43]. We found that self-compassion is seen by Canadian physiatrists as antithetical to the medical training they received. This is unfortunate, as training in self-compassion can help health care providers better self-care, improve compassion for others, and may be protective against burnout with moderate to large effect sizes [44], whilst Compassionate Mind Training may be particularly beneficial in addressing shame-based disorders associated with burnout [45].

### Strengths and Limitations

This is the first qualitative study examining the origins and sustaining factors of burnout in PM&R. Strengths include a large, diverse Canadian sample, rigorous methods, and varied team perspectives. Limitations include the majority of participants being full-time urban physiatrists from Ontario, a lack of regional analysis, and a cross-sectional design, which may miss formative early-career experiences and long-term patterns in burnout development. No PM&R residents being interviewed is a notable limitation because many of participants indicated origins of burnout date back to experiences in medical school and residency. We used Canadian PM&R leaders to identify participants, consistent with purposive sampling but potentially introducing selection bias. Self-reported interviews may also be affected by recall or social desirability biases. As a preliminary grounded theory, the framework is at an early stage, may not capture all nuances of burnout, and requires further empirical validation. Findings are specific to Canadian PM&R and may not generalize elsewhere.

### Future Directions

Future research should explore the views and experiences of PM&R residents and specifically seek to delve deeper into how intersectional factors influence burnout among physiatrists from marginalized backgrounds within Canada and elsewhere. Research should consider how aspects such as immigration status, ethnic minority status, religious beliefs, and sexual orientation intersect with burnout experiences. Marginalized physiatrists might face unique stressors related to their identity that impact their professional experiences differently than those of their peers. For instance, immigrants might deal with additional challenges related to cultural integration and professional validation, while members of minority ethnic or religious groups might encounter systemic biases or discrimination. It would also be very useful to explore how regional variation in normative organizational culture, health care systems, and wellness practices influences the experiences of physiatrists with respect to hierarchy, hidden curricula, bureaucracy, and burnout.

### Conclusions

The origins of burnout in Canadian physiatrists relate to a hierarchical performance paradox that appears to be seeded early in their careers. Budding Canadian physiatrists cite altruistic motivations for joining the specialty, but it is difficult to maintain compassion for oneself and others amidst a hidden curriculum that prioritizes academic elitism over compassionate care. Risk of burnout among Canadian physiatrists is described as peaking in residency but is also ever-present due to the emotionally demanding nature of the work for which they feel ill-prepared, fragmented health care systems, and societal bureaucracy around disability. Burnout can be a source of shame for Canadian physiatrists and is perceived as having the potential for severe professional repercussions. Canadian physiatrists value positive peer appraisal and emotional support (ie, a supportive community); this provides a potential inroad to addressing some of the factors that are responsible for burnout in the specialty.

## Appendix

Multimedia Appendix 1 Semistructured interview topic guide.

## Abbreviations

OR: odds ratio
PM&R: Physical Medicine and Rehabilitation

## Data Availability

Study data will be made available on reasonable request to the corresponding author.
