# Supplementary material for: Understanding the Origins and Factors of Burnout in Physical Medicine and Rehabilitation: Grounded Theory Analysis
Source: JMIR Rehabil Assist Technol. 2026 Feb 26;13:e80499. doi: 10.2196/80499 (PMC12982955; doi:10.2196/80499)
Supplement: Multimedia Appendix 1 [file rehab_v13i1e80499_app1.docx]

**Appendix A**

*Semi-Structured Interview Guide*

1. Can you please describe what compassion means to you?
   1. Can you describe whether/how you were taught about compassion?
2. Can you please describe what self-compassion means to you?
   1. (Probe about self-kindness vs self-judgement, common humanity vs isolation, mindfulness vs over-identification)
3. Can you please describe any strategies you use to cultivate/ maintain compassion for yourself/ others?
   1. (Probe about self-care strategies e.g., work-life balance, diet, sleep, exercise, stress management, meditation, time with friends and family)
   2. (Probe about Continuing Medical Education)
4. Can you please describe any factors you perceive that prevent you from being compassionate towards yourself?
5. Can you please describe any factors you perceive that prevent you from being compassionate towards others?
   1. (Probe about personal factors, interpersonal conflict, personality, role models, communication skills, past work/ life experiences
   2. (Probe about patient factors, complexity, diagnoses, impairments, disabilities, health behaviours, non-adherence, interpersonal conflict, complaints)
   3. (Probe about work factors, time of day, job environment, privacy, pagers/ interruptions, bed occupancy pressures, administrative burden)
6. Can you please describe how you use compassion as a Physiatrist and/or give an example?
   1. Can you please describe what you typically think or feel when you perceive that a person is suffering emotionally?
   2. Can you please describe what you typically do when you perceive a person is suffering emotionally?
   3. Can you please describe what you typically think or feel when you perceive a person is suffering physically?
   4. Can you please describe what you typically do when you perceive a person is suffering physically?
7. Can you please describe what compassion fatigue means to you?
   1. Can you describe whether/how you were taught about compassion fatigue?
   2. Please describe any experience you have with it personally?
   3. Please describe any strategies you use to prevent it?
   4. Please describe how, if at all, compassion fatigue might affect your ability to provide compassionate care as a Physiatrist?
8. Can you please describe a time where you felt compassion was lacking in care you or someone else provided in Physiatry?
   1. Why do you think this happened?
   2. What do you think might have helped prevent this from happening?
9. Can you please describe what burnout means to you?
   1. Can you describe whether/how you were taught about burnout?
   2. Please describe any experience you have with it personally?
   3. Please describe any strategies you use to prevent it?
10. Can you please describe your views on the emotional burden associated with working in Physiatry?
    1. Can you please describe how you deal with the emotional burden you experience working in Physiatry?
11. Can you please describe what you enjoy most about your work in Physiatry?
12. Can you please describe what you enjoy least about your work in Physiatry?
13. Can you please describe your best professional achievements?
14. Is there anything else you would like to add?
